# Interleukin-10 disrupts liver repair in acetaminophen-induced acute liver failure

**DOI:** 10.3389/fimmu.2023.1303921

**Published:** 2023-11-29

**Authors:** Katherine Roth, Jenna Strickland, Asmita Pant, Robert Freeborn, Rebekah Kennedy, Cheryl E. Rockwell, James P. Luyendyk, Bryan L. Copple

**Affiliations:** ^1^ Department of Pharmacology and Toxicology, Michigan State University, East Lansing, MI, United States; ^2^ Institute for Integrative Toxicology, Michigan State University, East Lansing, MI, United States; ^3^ Cell and Molecular Biology Program, Michigan State University, East Lansing, MI, United States; ^4^ College of Human Medicine, Michigan State University, East Lansing, MI, United States; ^5^ Pathobiology and Diagnostic Investigation, Michigan State University, East Lansing, MI, United States

**Keywords:** acetaminophen, acute liver failure, interleukin-10, inflammation, monocytes

## Abstract

**Introduction:**

Systemic levels of the anti-inflammatory cytokine interleukin 10 (IL-10) are highest in acetaminophen (APAP)-induced acute liver failure (ALF) patients with the poorest prognosis. The mechanistic basis for this counterintuitive finding is not known, as induction of IL-10 is hypothesized to temper the pathological effects of immune cell activation. Aberrant production of IL-10 after severe liver injury could conceivably interfere with the beneficial, pro-reparative actions of immune cells, such as monocytes.

**Methods:**

To test this possibility, we determined whether IL-10 levels are dysregulated in mice with APAP-induced ALF and further evaluated whether aberrant production of IL-10 prevents monocyte recruitment and/or the resolution of necrotic lesions by these cells.

**Results:**

Our studies demonstrate that in mice challenged with 300 mg/kg acetaminophen (APAP), a hepatotoxic dose of APAP that fails to produce ALF (i.e., APAP-induced acute liver injury; AALI), Ly6C^hi^ monocytes were recruited to the liver and infiltrated the necrotic lesions by 48 hours coincident with the clearance of dead cell debris. At 72 hours, IL-10 was upregulated, culminating in the resolution of hepatic inflammation. By contrast, in mice treated with 600 mg/kg APAP, a dose that produces clinical features of ALF (i.e., APAP-induced ALF; AALF), IL-10 levels were markedly elevated by 24 hours. Early induction of IL-10 was associated with a reduction in the hepatic numbers of Ly6C^hi^ monocytes resulting in the persistence of dead cell debris. Inhibition of IL-10 in AALF mice, beginning at 24 hours after APAP treatment, increased the hepatic numbers of monocytes which coincided with a reduction in the necrotic area. Moreover, pharmacologic elevation of systemic IL-10 levels in AALI mice reduced hepatic myeloid cell numbers and increased the area of necrosis.

**Discussion:**

Collectively, these results indicate that during ALF, aberrant production of IL-10 disrupts the hepatic recruitment of monocytes, which prevents the clearance of dead cell debris. These are the first studies to document a mechanistic basis for the link between high IL-10 levels and poor outcome in patients with ALF.

## Introduction

In severe cases of acetaminophen (APAP) overdose, acute liver injury rapidly progresses to acute liver failure (ALF), producing life threatening cardiac instability, hepatic encephalopathy, and multiorgan dysfunction syndrome (MODS) (1, 2). First line therapy for APAP overdose is N-acetyl cysteine (NAC), which is most efficacious when administered within 8 hours of APAP ingestion (3, 4). If NAC fails to limit the progression to ALF, supportive medical care and liver transplantation are the only remaining modes of therapy. Unfortunately, despite significant improvements to critical care medicine and emergency liver transplantation, mortality associated with ALF remains high (i.e., approximately 30%), underscoring the importance of identifying new therapeutic targets (2).

Components of innate immunity exacerbate early liver injury after APAP overdose in mice. Consequently, deficiency of the neutrophil chemokine, Cxcl1, or antibody-mediated depletion of neutrophils reduces blood biomarkers of hepatocyte injury in APAP-treated mice (5, 6). Moreover, deficiency in the anti-inflammatory cytokine, interleukin-10 (IL-10), enhances liver injury and inflammation in APAP-treated mice, whereas administration of recombinant IL-10 early after APAP overdose reduces liver injury (7–9). These findings have set current dogma that innate immunity is predominately detrimental to outcome after APAP overdose. As a result, it has been proposed that therapeutic intervention with exogenous IL-10 could improve outcomes in APAP overdose patients that have become refractory to standard care (9). Although this could be viewed as a game changer for critically ill patients, the prevailing belief that the innate immune system is solely harmful contrasts with findings from clinical studies where severe immune suppression is frequently observed in this patient population (10–12).

APAP-induced ALF patients with the poorest prognosis develop compensatory anti-inflammatory response syndrome (CARS), a condition characterized by severe systemic immune suppression (10, 13). Blood monocytes collected from ALF patients demonstrate a reduced capacity to phagocytose bacteria, produce lower levels of proinflammatory cytokines, and display features of myeloid-derived suppressor cells (MDSCs), including high level expression of the immune-suppressive, ligand-receptor pair, programmed death ligand 1 (PD-L1) and PD-1 (11, 14, 15). Moreover, high blood levels of the CARS-associated cytokine, IL-10, are an independent predictor of a poor outcome in these patients (10, 12). While the mechanistic basis for this seemingly paradoxical association is not known, these findings indicate that therapeutic intervention with IL-10 could be harmful to ALF patients. One mechanism by which high levels of IL-10 could negatively impact outcome in APAP overdose patients is by interfering with the beneficial, pro-reparative activities of innate immune cells.

Liver injury after APAP overdose stimulates the hepatic release of Ccl2, a chemokine that facilitates the recruitment of Ly6C^hi^ monocytes to the damaged liver in a Ccr2-dependent manner (16, 17). Once in the liver, these cells traffic to the necrotic foci where they become stimulated to phagocytose dead cell debris (17, 18). This process, along with proliferation of hepatocytes and nonparenchymal cells, facilitates repair of the damaged liver resulting in the restoration of hepatic function. Interventions that prevent the hepatic recruitment of monocytes, such as blockade of Ccr2, inhibit the clearance of dead cell debris, a key component of liver repair (16, 17). Because IL-10 is a potent anti-inflammatory cytokine, it is conceivable that high levels of IL-10 could interfere with certain pro-repair activities of immune cells, including resolution of necrotic lesions by monocytes. Interestingly, Bhushan and colleagues recently reported that necrotic cells persist in the livers of mice treated with a dose of APAP that produces clinically relevant ALF, including evidence of a coagulopathy, hepatic encephalopathy, and renal injury, a frequent component of MODS (19–22). Whether this is the result of amplified IL-10 levels, similar to APAP-induced ALF patients, however, was not investigated. Accordingly, in the present studies we tested the hypothesis that high levels of IL-10 in mice with APAP-induced ALF prevent the clearance of dead cell debris by monocytes.

## Materials and methods

### Animals and treatments

12-16-week-old male C57BL/6J or IL-10 reporter mice (B6.129S6-Il10^tm1/flv^/J; Jackson Laboratories) were used for all studies. Mice were housed in a 12-hour light/dark cycle under controlled temperature (18-21°C) and humidity. Food (Rodent diet; Harlan-Teklad) and water were allowed *ad libitum*.

Mice were fasted for 16 hours prior to the administration of 300 mg/kg APAP (Sigma-Aldrich), 600 mg/kg APAP, or sterile saline vehicle by intraperitoneal injection, as described previously (20). Fasting was initiated at approximately 5:00 PM and APAP was injected at approximately 9:00 AM the following day. In all studies, rodent diet was returned immediately after APAP challenge.

For IL-10 neutralization studies, mice were injected with 0.5 mg *InVivo*MAb anti-mouse IL-10 antibody (Bio X Cell, clone JES5-2A5) or 0.5 mg isotype control antibody (Innovative Research, Rat IgG) both by intraperitoneal injection at 24 hours after APAP treatment. For recombinant IL-10 studies, mice were injected with 5 μg recombinant mouse IL-10 (Biolegend, San Diego, CA) or sterile saline both by intraperitoneal injection at 24 hours after APAP challenge. All studies were approved by the Michigan State University Institutional Animal Care and Use Committee.

### Sample collection

Mice were anesthetized using Fatal-Plus Solution (Vortech Pharmaceuticals) or isoflurane. Blood was collected from the inferior vena cava and the livers were removed. A portion of each liver was fixed in 10% neutral-buffered formalin. The livers were embedded in paraffin, sectioned, and stained with hematoxylin and eosin. The area of necrosis was quantified as described by us previously (23). Briefly, 10 randomly chosen images were collected per liver section (2 sections per mouse taken from separate liver lobes) using a 20X objective. The areas of necrosis were outlined, and Image J was used to calculate the percent area of necrosis. Additional portions of the liver were homogenized in TRIzol Reagent (Thermo-Fisher Scientific) for RNA isolation or were snap-frozen in liquid nitrogen for sectioning and immunofluorescence staining.

### Immunofluorescence

Immunofluorescence was used to detect CD68 as described by us previously (23). Briefly, 8 μm sections were cut from frozen livers and fixed for 10 minutes in 4% formalin. The sections were then incubated in blocking buffer (10% goat serum in phosphate-buffered saline (PBS)) followed by incubation with rat anti-CD68 antibody (Bio-Rad) diluted 1:500. After washing, the sections were incubated with goat anti-rat secondary antibody conjugated to Alexa Fluor 594 (diluted 1:500, Thermo Fisher Scientific). To quantify the area of CD68 fluorescent staining, 10 randomly chosen images were collected per liver section using a 20X objective. The area of positive CD68 staining was then quantified using Image J. Proliferating cell nuclear antigen (PCNA) was detected by immunohistochemistry and used as a biomarker of hepatocyte proliferation. Hepatocytes were identified based upon their larger size relative to that of nonparenchymal cells and quantified as described by us previously (23).

### Luminex immunoassay

IL-10 protein levels were measured in blood serum samples by using the Bio-Plex Pro assay kit (Bio-Rad) according to manufacturer’s instructions. Bead fluorescent readings were obtained using a Luminex 200 system.

### Isolation of F4/80^+^ and LY6C^+^ cells from mouse livers

To isolate F4/80^+^ and Ly6C^+^ cells, livers from C57BL/6 mice were perfused and digested with collagenase (Collagenase H; Sigma-Aldrich), as described previously (24). After removal of hepatocytes by centrifugation, the nonparenchymal cells were centrifuged at 300 × *g* for 10 minutes. A total of 1 × 10^8^ nonparenchymal cells was resuspended in 60 μL of MACS Buffer (2.5 g bovine serum albumin, 0.416 g EDTA, and 500 mL phosphate-buffered saline) containing 12 μL biotinylated anti-F4/80 antibody or biotinylated anti-Ly6C antibody (Miltenyi Biotec, Bergisch Gladbach, Germany). The cell suspension was incubated for 10 minutes at 4°C and then washed by adding 10 mL of MACS buffer and centrifugation (300 × *g* for 10 minutes). Streptavidin microbeads (Miltenyi Biotec), diluted 1:10 in 60 μL of MACs buffer, were added to the nonparenchymal pellet. Cells were resuspended and incubated at 4°C for 10 minutes and then washed by adding 10 mL of MACS buffer and centrifugation (300 × *g* for 10 minutes). The pellet was resuspended with 500 μL MACS buffer and applied to MACS LS columns (Miltenyi Biotec). The column was rinsed three times with 3 mL MACS buffer. F4/80^+^ or Ly6C^+^ cells were collected by removing the column from the midiMACS Separator (Miltenyi Biotec) and rinsing the column with 5 mL of MACS buffer. Cell purity was confirmed by flow cytometry and was routinely greater than 94% as reported by us previously (25). RNA was immediately isolated from the cells by using the E.Z.N.A. Total RNA Kit I (Omega, Bio-tek, Norcross, GA) and real-time PCR was performed as detailed below.

### Flow cytometry

To isolate non-parenchymal cells, mouse livers were perfused and digested with collagenase (Collagenase H, Sigma Chemical Company) as described by us previously (26). Hepatocytes were removed by centrifugation (50 g for 2 minutes), and non-parenchymal cells were collected from the remaining solution by centrifugation at 300 g for 10 minutes. The non-parenchymal cells were washed and resuspended in FACs buffer (phosphate-buffered saline, 1% fetal bovine serum). The cells were then incubated with Fc blocking buffer (BD Biosciences; diluted 1:20) for 10 minutes at 4 °C, rinsed, and then pelleted by centrifugation at 300 g for 5 minutes. The cells were incubated with anti-F4/80 conjugated to Pacific Blue, anti-Ly6C conjugated to APC/Cy7, and anti-Ly6G conjugated to Percp/Cy5.5 for 30 minutes at 4°C. All antibodies were purchased from Biolegend. For studies with IL-10 GFP reporter mice, the following antibodies were used for flow cytometry: anti-Axl (APC), anti-CD45.1 (Alexa 488), anti-F4/80 (pacific blue), anti-Cx3cr1 (Alexa 700), anti-Ly6C (APC/Cy7), anti-Cd11b (PE), anti-Marco (APC), anti-Ccr2 (BV650), anti-PD-L1 (PerCp/Cy5.5), anti-CD3 (BV510), anti-CD4 (AF700), anti-NK1.1 (BV711), anti-CD8a (PerCP), and CD11c (BV711). All antibodies were purchased from Biolegend, except anti-Marco and anti-Axl, which were purchased from Invitrogen. The fixable dye, Zombie Aqua (Biolegend), was used to determine cell viability. Following incubation, cells were washed twice and fixed in formalin (Sigma) for 15 minutes at 4°C. The fixed cells were washed twice and resuspended in FACs buffer. Flow cytometry was conducted on an Attune NxT flow cytometer (Life Technologies). Attune NxT software (Life Technologies) was used to analyze the data.

### Real-time PCR

Total RNA was isolated from liver samples using TRIzol Reagent (Thermo-Fisher) and reverse transcribed into cDNA as described previously(24). Real-time PCR was performed on a QuantStudio 7 Flex Real-Time PCR System (Thermo-Fisher) using the iTaq Universal SYBR green Supermix (Bio-Rad). The following primer sequences were used: TNF-α: Forward-5’-AGGGTCTGGGCCATAGAACT-3’, Reverse-5’-CCACCACGCTCTTCTGTCTAC-3’; Cxcl1: Forward-5’-TGGCTGGGATTCACCTCAAG-3’, Reverse-5’-GTGGCTATGACTTCGGTTTGG-3’; Ccl2: Forward- 5’-CCTGCTGTTCACAGTTGCC-3’, Reverse- 5’-ATTGGGATCATCTTGCTGGT-3’; Il-10: Forward- 5’-TGTCAAATTCATTCATGGCCT-3’, Reverse- 5’-ATCGATTTCTCCCCTGTGAA-3’: Rpl13a: Forward- 5’-GACCTCCTCCTTTCCCAGGC-3’, Reverse- 5’-AAGTACCTGCTTGGCCACAA-3’; urokinase plasminogen activator (uPA): Forward-5’-GGGCTTGTTTTTCTCTGCAC-3’, Reverse-5’-GCCCCACTACTATGGCTCTG-3’.

### Microarray analysis

For reanalysis of microarray data from ALF patients, the.cel files were obtained from GEO database (accession number: GSE8075) and differential gene expression was determined using Transcriptome Analysis Console Software (ThermoFisher Scientific). Detailed patient information was reported previously (27). Upstream analysis was conducted using Ingenuity Pathway Analysis (Qiagen).

### Statistical analysis

Results are presented as the mean + SEM. Data were analyzed by a one-way or two-way Analysis of Variance (ANOVA) where appropriate. Data expressed as a percentage were transformed by arcsine square root prior to analysis. Comparisons among group means were made using the Student-Newman-Keuls test. The criterion for significance was *p* < 0.05 for all studies.

## Results

### Persistence of necrotic cell debris in the livers of mice with APAP-induced ALF

Bhushan and colleagues previously reported that dead cells persist in the livers of mice with APAP-induced ALF (AALF) (20). Because monocytes are critical for the clearance of dead cells from the liver (28), these findings suggested a defect in monocyte recruitment and/or function in ALF. To examine this further, we treated groups of mice with 300 mg/kg APAP, a hepatotoxic dose of APAP that fails to produce ALF (i.e., APAP-induced acute liver injury; AALI), or with 600 mg/kg APAP, a dose that produces clinical features of AALF, including coagulopathy (22), renal injury (21), and hepatic encephalopathy (19).

**Figure 1:**
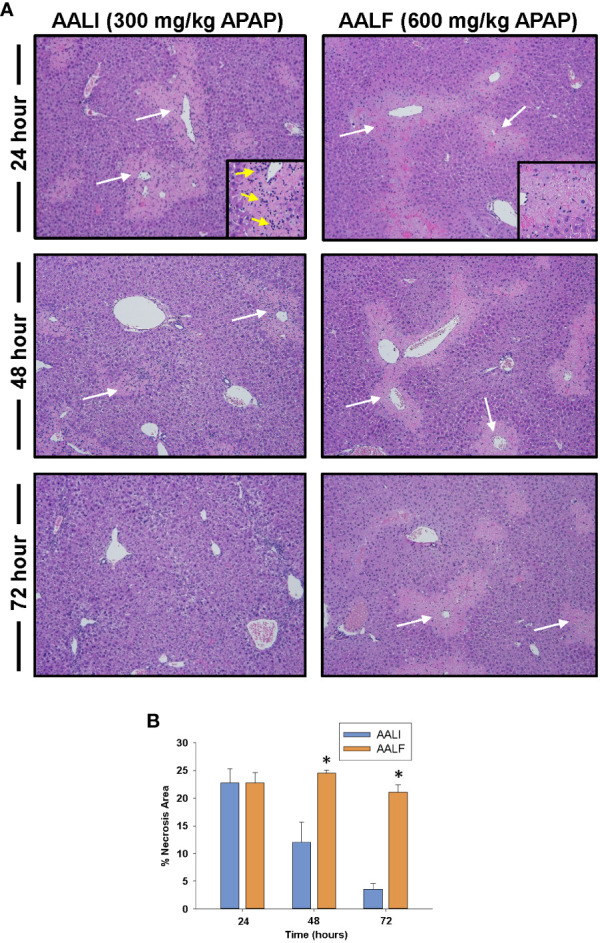
Liver injury and inflammation in APAP-treated mice. Mice were treated with either 300 mg/kg APAP (AALI) or 600 mg/kg APAP (AALF) for the indicated time. **(A)** Photomicrographs of H&E-stained liver sections. White arrows indicate areas of necrosis occurring in centrilobular regions. Higher power image of necrotic region in inset in 24 hour images. Arrows indicate evidence of inflammatory infiltrates. **(B)** Area of necrosis was quantified in sections of liver. *Significantly different from mice treated with 300 mg/kg APAP. All data are expressed as mean ± SEM; n = 5-10 mice per group.

As reported previously (20), liver necrosis peaked at approximately 24 hours in mice with AALI (**Figures 1A, B**). By 48 hours, extensive inflammatory infiltrates were evident within the necrotic foci (inset in **Figure 1A**, 24-hour AALI). The inflammatory cells, along with the necrotic cell debris, were largely cleared from the liver by 72 hours (**Figures 1A, B**). In mice with AALF, APAP produced a comparable initial hepatotoxic response (i.e., peak area of necrosis at 24 hours; **Figures 1A, B**), however, at this larger dose of APAP, the necrotic lesions were largely devoid of inflammatory cells (inset in **Figure 1A**, 24-hour AALF). Unlike AALI mice, the necrotic cells persisted in the liver at 72 hours, suggesting that the mechanisms driving dead cell clearance were impaired (**Figure 1B**) (17). While no deaths were noted in mice with AALI, in mice with AALF, 9 mice either died or were euthanized for humane reasons beyond 24 hours resulting in an overall mortality of 30% (n=30 mice with 9 deaths occurring at various times) by 72 hours. This mortality rate is similar to what was reported previously (20).

### Reduced numbers of myeloid cells in the livers of mice with AALF

**Figure 2:**
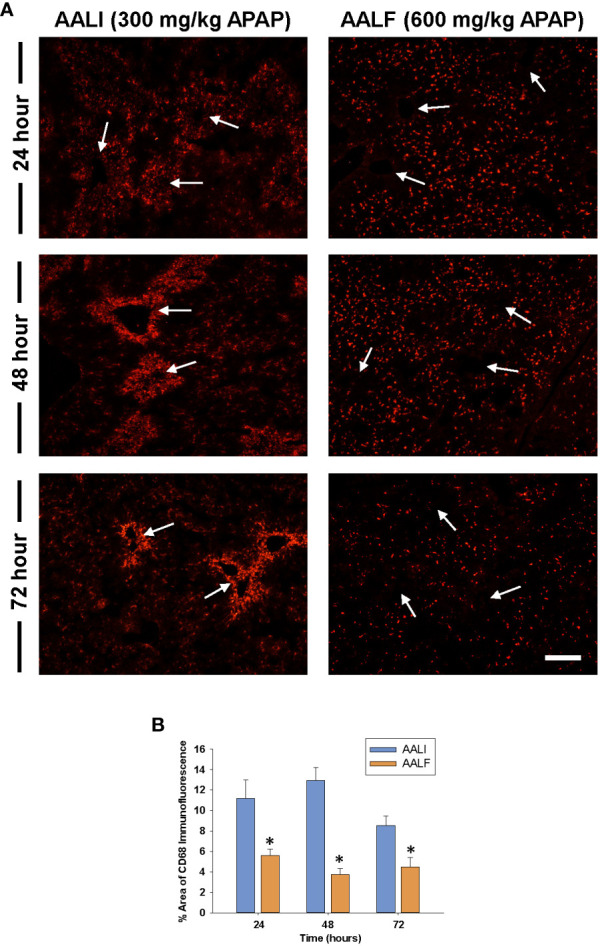
Accumulation of myeloid cells in the livers of mice treated with APAP. Mice were treated with either 300 mg/kg APAP (AALI) or 600 mg/kg APAP (AALF)for the indicated time. **(A)** Photomicrographs of CD68 immunofluorescent staining in liver sections. Positive staining appears red. Arrows indicate centrilobular regions. **(B)** The area of CD68 immunofluorescent staining was quantified in sections of liver. *Significantly different from mice treated with 300 mg/kg APAP. Data are expressed as mean ± SEM; n = 5 mice per group.

Because myeloid cells clear dead cell debris from the APAP-injured liver (28), we determined whether the numbers of these cells were impacted in the livers of AALF mice. To accomplish this, immunofluorescent staining was used to detect CD68 in sections of liver. CD68 is expressed at high levels by Ly6C^hi^ monocytes and Kupffer cells (29). Moreover, prior studies revealed that CD68^+^ myeloid cells accumulate in the livers of patients with APAP-induced ALF (18, 30, 31). By 24 hours in mice with AALI, CD68^+^ myeloid cells were present in the liver and more concentrated near centrilobular regions where liver injury occurred (**Figure 2A**, CD68 staining appears red; arrows indicate the location of central veins in centrilobular regions which were visible under conditions of overexposure). By 48 hours, CD68 staining was markedly increased in centrilobular regions which persisted at 72 hours (**Figure 2A**). In mice with AALF, fewer CD68^+^ myeloid cells were present in the livers at all time points examined (**Figures 2A, B**). Moreover, these cells were evenly distributed throughout the liver lobule and not concentrated within centrilobular regions (**Figures 2A, B**).

**Figure 3:**
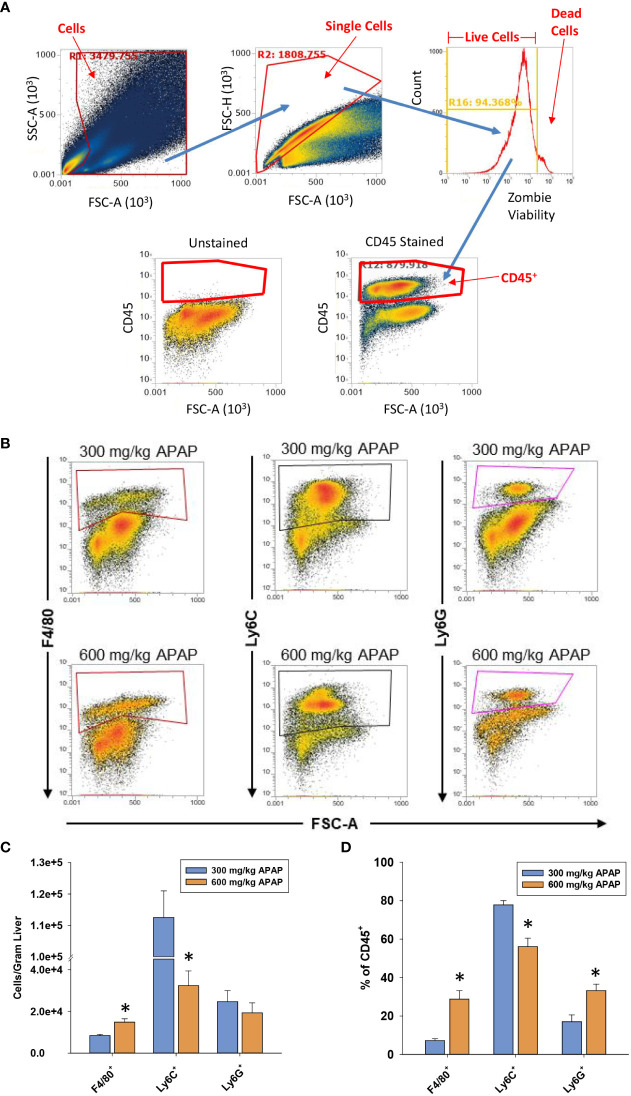
Hepatic myeloid cell accumulation in APAP-treated mice. Mice were treated with either 300 mg/kg APAP (AALI) or 600 mg/kg APAP (AALF)for the indicated time. **(A)** Gating for flow cytometry. **(B)** At 24 hours after APAP treatment, flow cytometry was used to detect F4/80^+^, Ly6C^+^, and Ly6G^+^ cells in the liver. Boxes indicate the positive gate. **(C)** Absolute cell counts and the percentage of each myeloid cell population within the larger CD45^+^ population by flow cytometry. *Significantly different from mice treated with 300 mg/kg APAP. All data are expressed as mean ± SEM; n = 5-10 mice per group.

Next, flow cytometry was used to better define the myeloid cell type(s) that were reduced in numbers in AALF mice. Gating for flow cytometry is shown in **Figure 3A**. As shown in **Figures 3B, C** the absolute number of Ly6G^+^ cells (i.e., neutrophils) in the liver was not different between mice with AALI and AALF, however these cells did comprise a larger percentage of the CD45 population in AALF mice (**Figure 3D**). The number of Ly6C^+^ cells (i.e., recruited monocytes; (32)) was markedly reduced in mice with AALF, whereas the number of F4/80^+^ cells (i.e., Kupffer cells and monocytes that had differentiated into macrophages, monocyte-derived macrophages (MDMs)) was modestly higher (**Figures 3B–D**). Collectively, these findings demonstrate that recruited monocytes, which clear dead cell debris from the APAP-injured liver, were decreased in numbers in mice with AALF.

### Increased levels of IL-10 in mice with AALF

**Figure 4:**
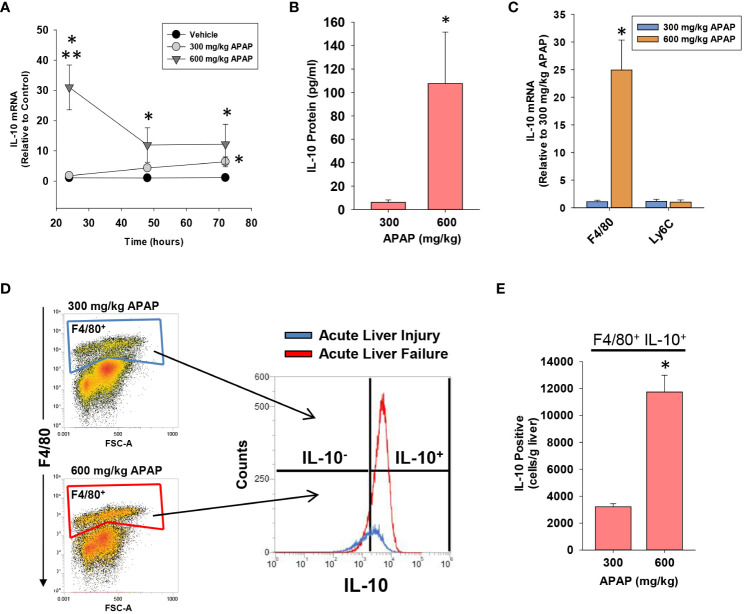
Hepatic and systemic levels of IL-10 in APAP-treated mice. Mice were treated with either 300 mg/kg APAP (AALI) or 600 mg/kg APAP (AALF). **(A)** At the indicated time, IL-10 mRNA levels were measured in the liver. **(B)** IL-10 protein was measured in serum at 72 hours after APAP treatment. **(C)** F4/80^+^ and Ly6C^+^ myeloid cells were isolated from the liver at 24 hours after treatment of mice with either 300 mg/kg APAP (AALI) or 600 mg/kg APAP (AALF), and IL-10 mRNA levels were measured. **(D)** Nonparenchymal cells were isolated from the livers of IL-10 reporter mice treated 24 hours earlier with 300 mg/kg APAP (AALI) or 600 mg/kg APAP (AALF). Flow cytometry was used to identify IL-10 expressing F4/80^+^ cells. Gate for F4/80^+^ cells indicated in the density plots. Representative histogram of IL-10 expression (i.e., GFP^+^) in F4/80^+^ cells. **(E)** Quantification of the number of F4/80^+^ cells expressing IL-10 in the liver from flow cytometry. *Significantly different from mice treated with 300 mg/kg APAP. Data are expressed as mean ± SEM; n = 4-5 mice per group.

High levels of IL-10 are associated with a worse outcome in ALF patients (10). Similar to these clinical findings, in mice with AALF, hepatic mRNA levels of IL-10 were increased by 24 hours and remained elevated at 72 hours (**Figure 4A**). Importantly, elevated mRNA levels were matched by increased IL-10 protein in the serum (**Figure 4B**). By contrast, in mice with AALI, IL-10 mRNA levels were not elevated until 72 hours after APAP treatment (**Figures 4A, B**). To determine whether myeloid cells were a source of IL-10, we purified F4/80^+^ myeloid cells (i.e., Kupffer cells and MDMs) and Ly6C^+^ myeloid cells (i.e., recruited monocytes) from the livers of AALF mice at 24 hours after APAP treatment and IL-10 mRNA levels were quantified. As shown in **Figure 4C**, IL-10 mRNA levels were greater in F4/80^+^ cells isolated from AALF mice but were not different between Ly6C^+^ cells isolated AALI and AALF mice (**Figure 4C**). We next confirmed these findings by using IL-10 reporter mice that express green fluorescent protein (GFP) under control of the IL-10 promoter. As shown in **Figures 4D, E**, greater numbers of GFP-expressing F4/80^+^ cells were detected in the livers of IL-10 reporter mice with AALF.

**Figure 5:**
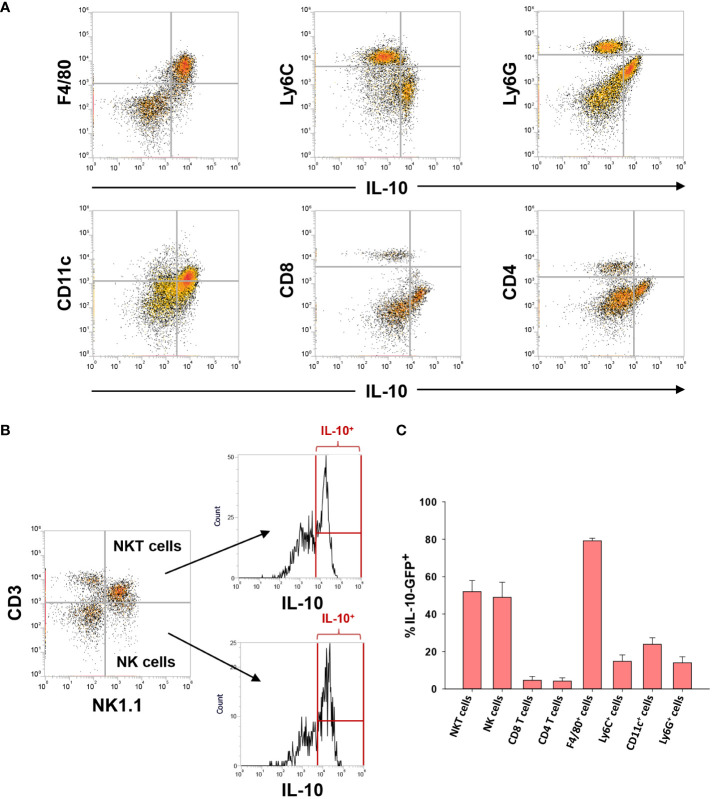
IL-10 expressing (GFP^+^) cells in the livers of mice with AALF. **(A, B)** Representative dot plots of the indicated cell types expressing IL-10. **(C)** Percentage of each immune cell type expressing IL-10 in AALF.

Next, we determined whether other immune cell types express IL-10 in the livers of mice with AALF. As shown in **Figure 5**, IL-10 (GFP) was detected in all immune cell types evaluated. Compared to other immune cell populations, however, a larger fraction of NKT cells, NK cells, and F4/80^+^ cells were positive for IL-10 in AALF mice (**Figure 5C**).

### Immunophenotyping of IL-10^+^ F4/80^+^ cells in mice with AALF

**Figure 6:**
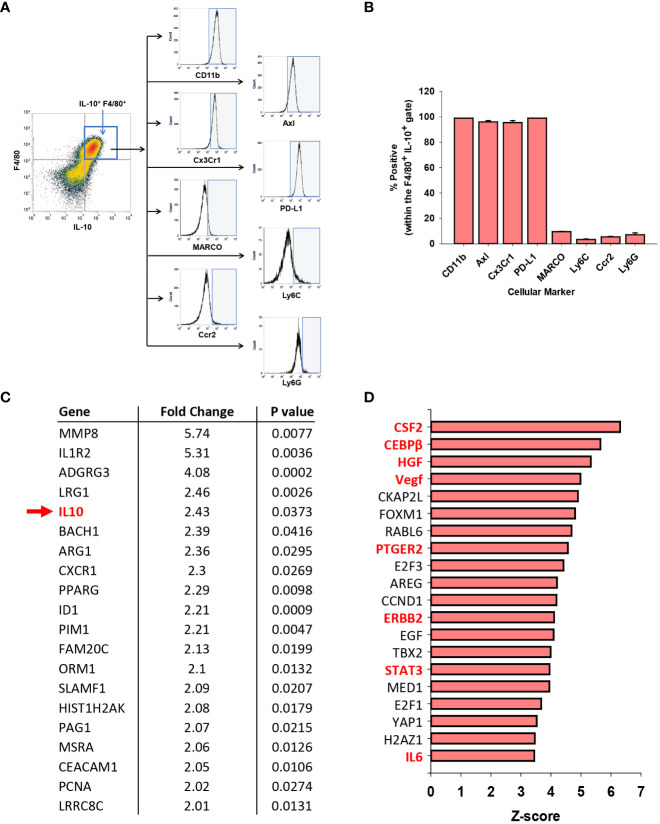
Immune suppressive phenotype of myeloid cells in APAP induced ALF. **(A)** Mice were treated with 600 mg/kg APAP. After 24 hours, flow cytometry was used to detect IL-10 expressing F4/80^+^ cells, indicated by the blue box. Representative histograms of IL-10^+^ F4/80^+^ cells expressing the indicated marker (x-axis). Positive staining indicated by the blue shaded box. **(B)** Quantification of the flow cytometry in **(A)** Data are expressed as mean ± SEM; n = 4 mice per group. **(C)** Analysis of GEO dataset accession number GSE8075, containing gene array analysis of monocytes isolated from patients with APAP-induced ALF that survived and patients with APAP induced ALF that died. Fold change represents ALF patients that died relative to those that survived (e.g., IL-10 mRNA levels 2.43-fold higher in patients that died; indicated with a green arrow and highlighted green). **(D)** Ingenuity pathway analysis was used to identify upstream regulators in the GSE8075 GEO dataset. Regulators associated with MDSCs are highlighted in red. (n=6 patient samples per group).

Clinical studies have demonstrated that circulating monocytes express markers of MDSCs in ALF patients with the worst outcomes (11, 33). Therefore, we determined whether the hepatic, IL-10-expressing F4/80^+^ cells (i.e., GFP^+^) similarly expressed markers of MDSCs in AALF mice. Immunophenotyping by flow cytometry revealed that these cells expressed CD11b, PD-L1, Cx3Cr1, and Axl consistent with an MDSC-like phenotype (**Figures 6A, B**). Levels of additional markers commonly associated with MDSCs, however, including MARCO, Ly6C, Ccr2, and Ly6G, were only detected in a small percentage of IL-10-expressing F4/80^+^ cells (**Figures 6A, B**).

Moore and colleagues previously performed gene array analysis on purified peripheral blood monocytes from patients with APAP-induced ALF (27). The analysis included 6 patients with AALF that spontaneously survived and 6 patients with AALF that either died or received a liver transplant (27). We reanalyzed this publicly available data set to determine whether IL-10 mRNA levels and levels of other MDSC-associated genes were differentially expressed between these two ALF patient populations.

As shown in **Supplemental Table 1**, 154 mRNAs were increased, while 37 mRNAs were decreased in blood monocytes from ALF patients that died when compared to those that survived. Many of these mRNAs, including IL-10 (indicated with a green arrow), have been associated with MDSCs (**Figure 6C**) (34, 35). Next, Ingenuity Pathway Analysis was used to identify potential upstream regulators that predict the observed mRNA changes. IPA identified several regulators as potential drivers of the observed transcriptional changes occurring in ALF patients that died (**Figure 6D**). Interestingly, many of these regulators have been linked to the differentiation, expansion, and/or activation of MDSCs (**Figure 6D**; MDSC drivers highlighted in red) (34, 35).

### Modulation of IL-10 levels impact myeloid cell accumulation and necrotic lesion size in mice treated with APAP

Our findings indicate that myeloid cell accumulation in the liver is disrupted in mice with AALF (**Figures 1**-**3**). Because of the potent immune inhibitory properties of IL-10, we tested the hypothesis that IL-10 contributes to this defect. To examine this, we utilized a loss of function approach (i.e., IL-10 neutralization in AALF mice) and a gain of function approach (i.e., injection of recombinant IL-10 into AALI mice). Prior studies demonstrated that IL-10 knockout mice develop markedly greater liver injury after APAP overdose (7). Accordingly, to avoid impacts on APAP-induced liver injury, we treated mice with IL-10 neutralizing antibody or recombinant IL-10 protein beginning at 24 hours after APAP treatment, a time where hepatocyte injury is complete (i.e., no additional increase in necrosis; **Figure 1**) and levels of hepatic glutathione, which detoxify the toxic APAP metabolite, N-acetyl-*p*-benzoquinone imine (NAPQI), have been restored (36).

**Figure 7:**
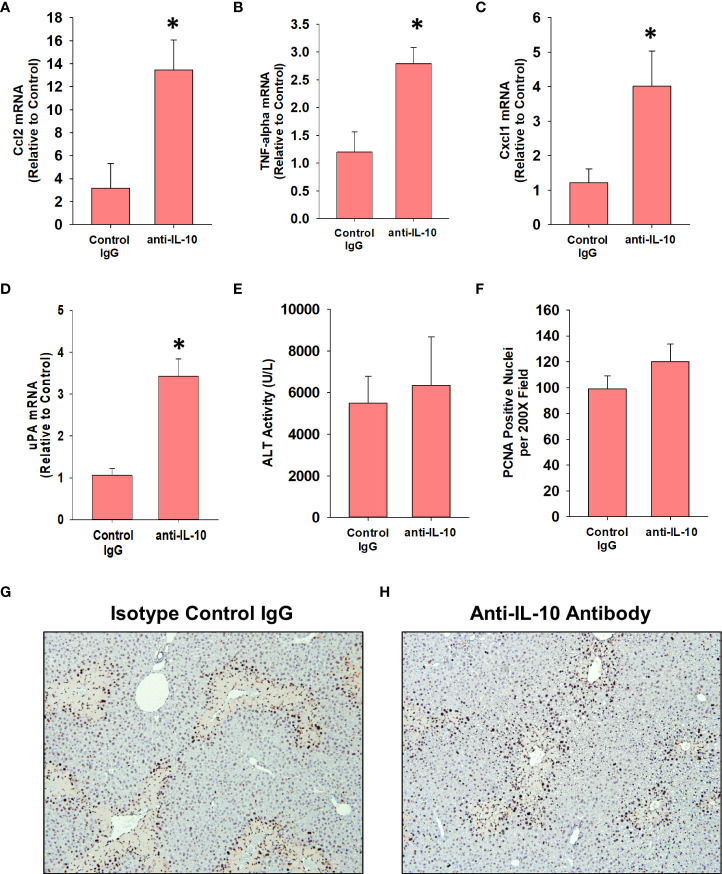
Impact of IL-10 neutralization in mice with APAP-induced ALF. Mice were treated with 600 mg/kg APAP (AALF) followed by treatment with control IgG or anti-IL-10 antibody 24 hours later. Livers were collected 72 hours after APAP treatment. **(A-D)** mRNA levels of the indicated protein were quantified in the liver by real-time PCR. **(E)** Serum activity of ALT. **(F)** The number of PCNA positive cells was quantified in sections of liver. Representative photomicrographs of immunohistochemistry staining for PCNA in liver sections from AALF mice treated with **(G)** isotype control IgG or **(H)** anti-IL-10 antibody. Positive staining appears dark brown. *Significantly different from mice treated with 600 mg/kg APAP and control IgG. All data are expressed as mean ± SEM; n = 5 mice per group.

**Figure 8:**
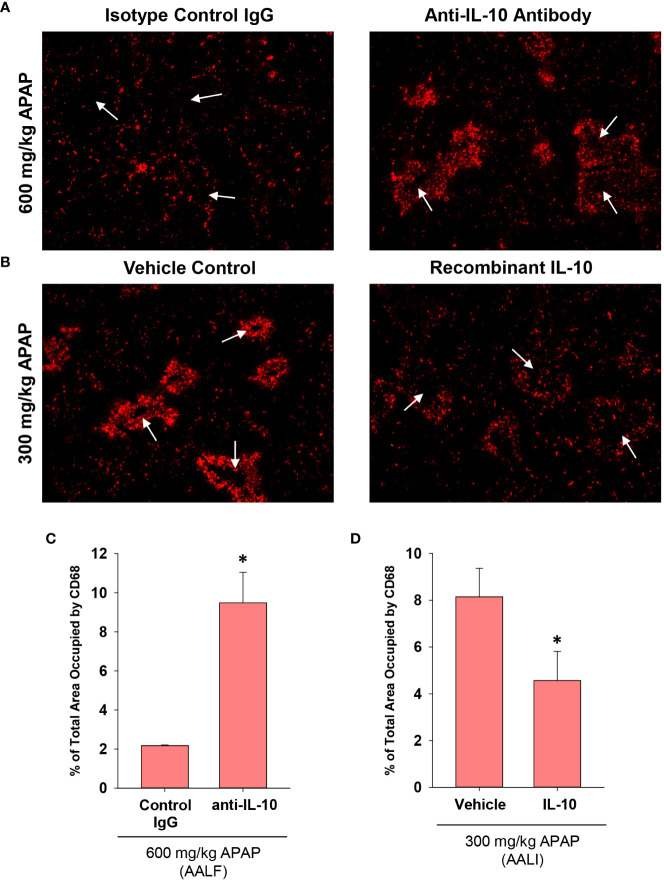
Impact of IL-10 on inflammation in mice treated with APAP. **(A)** Mice were treated with 600 mg/kg APAP (AALF) followed by treatment with control IgG or anti-IL-10 antibody 24 hours later. Livers were collected 72 hours after APAP treatment. CD68 was detected by immunofluorescence (red staining) in sections of liver. Arrows indicate centrilobular regions. **(B)** Mice were treated with 300 mg/kg APAP (AALI) followed by treatment with either vehicle or 5 mg recombinant IL-10 24 hours later. Livers were collected 48 hours after APAP treatment. CD68 was detected by immunofluorescence (red staining) in sections of liver. Arrows indicate centrilobular regions. **(A, B)** Representative photomicrographs from an n = 5 mice per group. **(C, D)** The area of CD68 staining was quantified in whole liver sections. n = 5 mice per group. *Significantly different at p<0.05.

Treatment of AALF mice with IL-10 neutralizing antibody increased mRNA levels of the pro-inflammatory cytokines, Ccl2, TNF-α, and Cxcl1 when compared to AALF mice treated with isotype control, demonstrating the efficacy of the IL-10 neutralizing antibody (**Figures 7A–C**). We also evaluated the impact of IL-10 neutralization on the levels of urokinase plasminogen activator (uPA) which converts the zymogen plasminogen into the fibrinolytic enzyme, plasmin. The rationale for this is that we demonstrated previously that inhibition of plasmin activity reduced monocyte-dependent clearance of dead cells from the livers of AALI mice and it is conceivable that diminished levels of uPA may contribute to the defective clearance of dead cell debris (18). As shown in **Figure 7D**, neutralization of IL-10 increased uPA mRNA levels in the livers of AALF mice. IL-10 neutralizing antibody, however, did not affect ALT activity in the serum (**Figure 7E**) or the number of PCNA positive cells in the liver (**Figures 7F–H**). In AALF mice treated with isotype control antibody, CD68^+^ myeloid cells were distributed throughout the liver lobule similar to our earlier findings (**Figure 8A**). Treatment of these mice with IL-10 neutralizing antibody, however, increased the numbers of CD68^+^ myeloid cells with a greater concentration in centrilobular regions (**Figures 8A, C**). Notably, the pattern of CD68^+^ immunostaining observed in these mice resembled that in AALI (**Figures 2A**, **8B**). Treatment of AALI mice with recombinant IL-10, on the other hand, reduced the numbers of CD68^+^ cells particularly in centrilobular regions (**Figures 8B, D**) producing a pattern of CD68 immunostaining similar to that observed in AALF mice (**Figures 2A**, **8A**).

**Figure 9:**
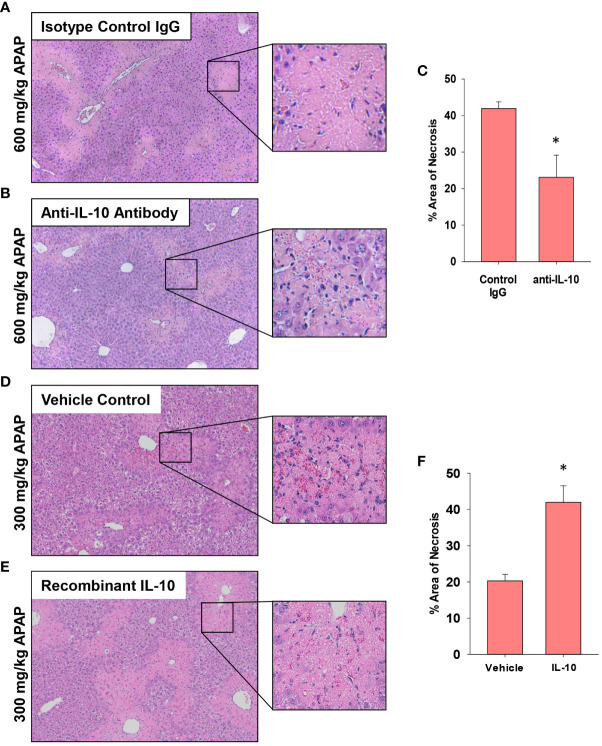
Impact of IL-10 on liver histology in mice treated with APAP. **(A–C)** Mice were treated with 600 mg/kg APAP (AALF) followed by treatment with control IgG or anti-IL-10 antibody 24 hours later. Livers were collected 72 hours after APAP treatment. **(D–F)** Mice were treated with 300 mg/kg APAP (AALI) followed by treatment with vehicle or 5 mg recombinant IL-10 24 hours later. Livers were collected 48 hours after APAP treatment. Representative photomicrographs of hematoxylin and eosin-stained liver sections from mice treated with **(A)** 600 mg/kg APAP (AALF) and control IgG, **(B)** 600 mg/kg APAP (AALF) and anti-IL-10 antibody, **(D)** 300 mg/kg APAP (AALI) and vehicle or **(E)** 300 mg/kg APAP (AALI) and recombinant IL-10. **(C, F)** The area of necrosis was quantified in the indicated liver sections. n = 5 mice per group. *Significantly different at p<0.05.

Next, because recruited monocytes clear dead cell debris from the APAP-injured liver, we determined whether increasing or decreasing IL-10 levels impacted lesion size. As in **Figure 8**, the indicated treatments (i.e., antibody or recombinant protein) were initiated at 24 hours, a time when peak injury had occurred (**Figure 1B**). Therefore, differences in the area of necrosis beyond 24 hours would result from changes to the mechanisms that clear dead cell debris. In AALF mice, neutralization of IL-10 increased histological evidence of inflammatory cells within the necrotic foci (**Figures 9A, B**) consistent with the CD68 immunostaining (**Figure 8A**). Further, IL-10 neutralizing antibody reduced the area of necrosis compared to isotype control treated mice (**Figure 9C**), suggesting that an increase in number of myeloid cells (**Figure 8**) resulted in an increase in the clearance of dead cell debris. Similar to these findings, pharmacological elevation of IL-10 in AALI mice decreased evidence of inflammatory cells within the necrotic lesions while increasing the area of necrosis (**Figures 9D–F**).

## Discussion

Toxicant-induced liver injury stimulates the synthesis and release of chemokines that recruit immune cells, including monocytes, to the liver. As the injury resolves, IL-10 is released to terminate the inflammatory response thereby preventing immune-mediated exacerbation of the injury. Evidence in support of this has demonstrated that deficiency of IL-10 (i.e., IL-10 knockout mice) exacerbates proinflammatory cytokine production and liver injury in APAP treated mice (7). More recently, it was reported that treatment of AALI mice with exogenous IL-10, beginning at 2 hours after APAP treatment, reduces liver injury (9). While these findings have driven current dogma that IL-10 is protective in experimental liver injury, they contrast with clinical findings where high levels of IL-10 are an independent predictor of a poor outcome in ALF patients (10). The mechanistic basis for this paradoxical association is not fully known, however, it has been proposed that high levels of IL-10 may prevent the clearance of nosocomial infections leading to sepsis (10). Investigations into this possibility, however, have been unable to demonstrate an association between high levels of IL-10 and increased risk of sepsis, suggesting that IL-10 impacts additional processes critical for recovery from ALF (10). Our current study provides an alternative explanation for these findings and reveals that high levels of IL-10 interfere with hepatic monocyte recruitment.

In mice with AALI, CD68^+^ myeloid cells began to concentrate within centrilobular regions of the liver where APAP produces injury (**Figures 1**, **2**). Coincident with the accumulation of these cells, dead cell debris was cleared from the liver (**Figures 1**, **2**) (17). By contrast, in mice with AALF, the numbers of CD68^+^ myeloid cells were markedly lower at all time-points examined, and these cells were largely restricted to uninjured regions (**Figure 2**). Immunophenotyping of the hepatic myeloid cell populations demonstrated a reduction in the numbers of Ly6C^+^ monocytes in the livers of AALF mice, indicating a defect in the recruitment of these cells from the systemic circulation (**Figure 3**). It was previously reported that manipulations which prevent the hepatic recruitment of monocytes (i.e., Ccr2 knockout mice) inhibit the clearance of necrotic cell debris from the APAP-injured liver (16, 17, 32). Consistent with these findings, our studies demonstrated that a paucity of Ly6C^+^ monocytes in the livers of AALF mice coincided with a persistence of dead cell debris (**Figures 1**, **2**). Collectively, these findings suggest that a failure of monocyte recruitment to the liver during AALF may prevent the clearance of necrotic cells. While it could be argued that the persistence of necrosis resulted from ongoing hepatocyte death that was compensated for by hepatocyte proliferation, this is unlikely in light of findings by Bhushan and colleagues demonstrating that hepatocyte proliferation is also markedly reduced in mice with AALF (20). Therefore, the persistence of the necrotic foci most likely resulted from a failure in the mechanisms controlling the clearance of dead cells. It would be difficult to assess whether there is a similar reduction in the hepatic accumulation of monocytes in the livers of patients with ALF. Although, it has been reported that ALF patients with the worst outcomes have reduced numbers of circulating monocytes when compared to ALF patients that recover (27).

Mechanistically, our studies indicate that early induction of IL-10 (**Figure 4A**) contributes to the defect in hepatic monocyte recruitment in AALF mice. In support of this, treatment of AALF mice with IL-10 neutralizing antibody increased monocyte numbers in the liver (**Figure 8A**), which coincided with a decrease in the area of necrosis, suggesting that the recovery of monocyte recruitment restored, in part, dead cell clearance (**Figures 9A, B**). The reduction in necrotic area in AALF mice treated with IL-10 neutralizing antibody cannot be attributed to a decrease in the severity of liver injury, as the antibody treatments were not initiated until 24 hours after APAP overdose, a time where hepatocyte injury is complete (i.e., no additional increase in necrosis; **Figure 1**) and levels of hepatic glutathione, which detoxify APAP, have been fully restored (36). Moreover, as mentioned above, a complete loss of IL-10 (i.e., IL-10 knockout mice) enhances rather than reduces liver injury (7). In further confirmation of the impact of IL-10 on liver repair, we demonstrated that pharmacological elevation of IL-10 in AALI mice, beginning at 24 hours after APAP treatment, reduced hepatic monocyte recruitment while increasing the area of necrosis (**Figures 8**, **9**). These findings indicate that high levels of IL-10 prevent the recruitment of monocytes to the liver which inhibits the clearance of dead cells. As discussed earlier, it was recently reported that treatment of AALI mice with exogenous IL-10, early after APAP overdose (i.e., 2 hours), attenuated liver injury. While this approach might similarly reduce liver injury in APAP overdose patients, it would require administration soon after the overdose which is a time where NAC is well established to be safe and highly efficacious. Moreover, our studies indicate that initiation of IL-10 therapy, after liver injury has occurred, could disrupt liver repair potentially resulting in a worse outcome.

Despite the reduction in necrotic area, AALF mice treated with either IL-10 neutralizing antibody or isotype control antibody were similarly moribund by 72 hours and were euthanized, indicating that the restoration of monocyte recruitment alone was insufficient to fully reverse the ALF pathology. One possible reason for this may be that neutralization of IL-10 failed to restore hepatocyte proliferation in AALF mice (**Figure 7E**). As discussed, Bhushan and colleagues reported previously that hepatocyte proliferation is markedly reduced in mice with AALF, and that this is reversed by pharmacological blockade of GSK-3β (37). Restoration of hepatocyte proliferation through GSK-3β inhibition, however, did not recover dead cell clearance and similar to our studies, was unable to improve survival (37). Therefore, it is possible that restoration of hepatocyte proliferation along with dead cell clearance mechanisms may be needed to promote a full recovery of liver function in ALF.

The mechanism by which neutralization of IL-10 enhanced monocyte recruitment to the liver was not fully uncovered from our studies, however, it may involve changes to the levels of the monocyte chemokine, Ccl2 or the fibrinolytic enzyme, uPA. Ccl2 is released after liver injury and binds to the Ccr2 receptor on circulating monocytes which triggers their recruitment towards sites of injury (16, 17). In our studies, neutralization of IL-10 increased hepatic Ccl2 levels (**Figure 6A**) which may have contributed to the enhanced recruitment of myeloid cells to the liver. A second mechanism by which IL-10 may prevent dead cell clearance from the liver is through inhibition of fibrinolysis. We previously showed that the fibrinolytic enzyme, plasmin, is critical for the clearance of dead cells from the livers of mice with AALI (18). The protease uPA cleaves the zymogen plasminogen to generate plasmin. Interestingly, prior studies demonstrated that IL-10 reduces uPA levels in cultured monocytes (38). Consistent with this *in vitro* finding, our studies show that neutralization of IL-10 in mice with AALF increased uPA levels suggesting that IL-10 may impact dead cell clearance by interfering with fibrinolysis. Additional studies are needed, however, to fully investigate these possibilities.

Increased numbers of circulating MDSCs have been reported in patients with ALF. Our studies complement these findings and indicate further that these cells are a potential source of IL-10 (**Figure 5C**). Notably, levels of IL-10 were greatest in circulating monocytes from patients with ALF that died or were referred for a liver transplant. Moreover, several mRNAs were increased in these cells that are associated with MDSCs, and ingenuity pathway analysis identified a number of upstream regulators that could contribute to the generation of these cells. Because of these findings, we determined whether the IL-10-expressing, F4/80^+^ macrophages in the livers of mice with AALF also expressed markers of MDSCs. Interestingly, these cells expressed several proteins frequently associated MDSCs, including PD-L1, CD11b, Axl, and Cx3Cr1 (**Figure 5**) (35). They did not, however, express Ly6C, a key marker associated with monocytic MDSCs (35). Therefore, the IL-10 producing cells are either Kupffer cells or may have arisen from circulating MDSCs. Studies have shown that MDSCs recruited to the tumor microenvironment mature into tumor-associated macrophages that lose expression of Ly6C, gain expression of F4/80 and Cx3Cr1, and maintain expression of IL-10 and PD-L1. Although additional studies are needed to investigate this, it is possible that in AALF, circulating MDSCs accumulate in the liver and mature into F4/80^+^ macrophages with an immune suppressive phenotype, similar to what occurs in solid tumors. Consistent with this possibility, our studies demonstrated that there were nearly twice as many F4/80^+^ myeloid cells in the livers of mice with AALF when compared to AALI mice (**Figure 3C**). Additional studies are needed, however, to better define the source of these immune suppressive cells. Similarly, additional follow up is needed to evaluate the importance of NKT and NK cells as a source of IL-10 in AALF, as our studies show that a high percentage of these cells produce IL-10 in the liver during AALF (**Figure 5**).

Collectively, our findings demonstrate that IL-10 dysregulation is effectively recapitulated in mice treated with a high dose APAP that produces ALF. As such, this experimental setting provides a novel platform to interrogate mechanisms of immune dysregulation in ALF and to identify new therapeutic interventions. Further, by using this approach, our studies identified IL-10 as a central player in monocyte dysregulation in ALF, challenging the long-held belief that IL-10 is only hepatoprotective in APAP-induced ALF and providing a mechanism to explain the paradoxical association between high levels of IL-10 and poor outcome in ALF patients.

## Data availability statement

The datasets presented in this study can be found in online repositories. The names of the repository/repositories and accession number(s) can be found in the article/**Supplementary Material**.

## Ethics statement

Ethical approval was not required for the study involving humans in accordance with the local legislation and institutional requirements. Written informed consent to participate in this study was not required from the participants or the participants’ legal guardians/next of kin in accordance with the national legislation and the institutional requirements. The animal study was approved by Institutional Animal Care and Use Committee, Michigan State University. The study was conducted in accordance with the local legislation and institutional requirements.

## Author contributions

KR: Conceptualization, Data curation, Formal analysis, Investigation, Writing – review & editing. JS: Conceptualization, Data curation, Formal analysis, Investigation, Writing – review & editing. AP: Conceptualization, Data curation, Formal analysis, Investigation, Writing – review & editing. RF: Data curation, Formal analysis, Investigation, Writing – review & editing. RK: Data curation, Formal analysis, Investigation, Writing – review & editing. CR: Data curation, Formal analysis, Investigation, Supervision, Writing – review & editing. JL: Conceptualization, Data curation, Formal analysis, Funding acquisition, Investigation, Supervision, Writing – review & editing. BC: Conceptualization, Data curation, Formal analysis, Funding acquisition, Investigation, Supervision, Writing – original draft.
