# Supplementary material for: Interleukin-10 disrupts liver repair in acetaminophen-induced acute liver failure
Source: Front Immunol. 2023 Nov 29;14:1303921. doi: 10.3389/fimmu.2023.1303921 (PMC10716295; doi:10.3389/fimmu.2023.1303921)
Supplement: Supplementary file 1 [file DataSheet_1.pdf]

Supplemental Table 1: Gene Expression Changes in ALF Patients that Died Relative to ALF Patients that Survived.

Column1	Column2	Column3	Column4	Column5	Column6	Column7	Column8
ID	ALF: Died Avg (log2)	ALF: Live Avg (log2)	Fold Change	P-val	FDR P-val	Gene Symbol	Description
17078592	5.68	2.64	8.21	0.0109	0.9999	FABP4	fatty acid binding protein 4, adipocyte
16860971	6.88	4.29	6.01	0.0057	0.9999	HAMP	hepcidin antimicrobial peptide
16743686	5.73	3.21	5.74	0.0077	0.9999	MMP8	matrix metallopeptidase 8
16883624	8.52	6.12	5.31	0.0036	0.9999	IL1R2	interleukin 1 receptor, type II
16819563	7.77	5.74	4.08	0.0002	0.9999	ADGRG3	adhesion G protein-coupled receptor G3
17016363	3.9	1.96	3.84	0.0059	0.9999	HIST1H3B	histone cluster 1, H3b
16850517	6.73	4.86	3.66	0.0157	0.9999	NDC80	NDC80 kinetochore complex component
16996722	6.79	4.96	3.55	0.0353	0.9999	CENPK	centromere protein K
16982024	7.51	5.7	3.51	0.0050	0.9999	CENPU	centromere protein U
17016499	6.6	4.81	3.44	0.0050	0.9999	HIST1H1B	histone cluster 1, H1b
16798553	3.76	1.98	3.43	0.0037	0.9999	MIR4509-1; MIR4509-2; MIR4509-3	microRNA 4509-1; microRNA 4509-2; microRNA 4509-3
16805940	3.76	1.98	3.43	0.0037	0.9999	MIR4509-1; MIR4509-2; MIR4509-3	microRNA 4509-1; microRNA 4509-2; microRNA 4509-3
16806388	3.76	1.98	3.43	0.0037	0.9999	MIR4509-1; MIR4509-2; MIR4509-3	microRNA 4509-1; microRNA 4509-2; microRNA 4509-3
16883715	5.85	4.15	3.26	0.0028	0.9999	IL18R1	interleukin 18 receptor 1
17101876	4.48	2.79	3.22	0.0010	0.9999	SCML1	sex comb on midleg-like 1 (Drosophila)
16979389	6.4	4.73	3.18	0.0154	0.9999	MAD2L1	MAD2 mitotic arrest deficient-like 1 (yeast)
17016403	7.13	5.48	3.13	0.0091	0.9999	HIST1H3G	histone cluster 1, H3g
16752185	6.98	5.34	3.12	0.0156	0.9999	METTL7B	methyltransferase like 7B
16971573	5.53	3.91	3.08	0.0153	0.9999	MND1	meiotic nuclear divisions 1
16807605	4.39	2.78	3.06	0.0136	0.9999	OIP5	Opa interacting protein 5
17016356	3.59	1.99	3.02	0.0018	0.9999	HIST1H1A	histone cluster 1, H1a
17008341	5.27	3.68	3.02	0.0014	0.9999	DAAM2	dishevelled associated activator of morphogenesis 2
16702705	3.71	2.12	3.01	0.0169	0.9999	OLAH	oleoyl-ACP hydrolase
17045198	6.97	5.39	2.99	0.0014	0.9999	ANLN	anillin actin binding protein
16970563	4.75	3.19	2.95	0.0052	0.9999	PLK4	polo-like kinase 4
16747014	6.34	4.81	2.88	0.0017	0.9999	RAD51AP1	RAD51 associated protein 1
16960054	5.65	4.14	2.86	2.06E-05	0.3304	PCOLCE2	procollagen C-endopeptidase enhancer 2
16901957	6.43	4.92	2.85	0.0498	0.9999	CKAP2L	cytoskeleton associated protein 2-like
17084904	5.6	4.12	2.79	0.0012	0.9999	MELK	maternal embryonic leucine zipper kinase
17005603	9.28	7.81	2.76	0.0258	0.9999	HIST1H2BI	histone cluster 1, H2bi
16832907	5.37	3.91	2.75	0.0422	0.9999	DPRXP4	divergent-paired related homeobox pseudogene 4
16799637	4.63	3.2	2.7	0.0017	0.9999	RAD51	RAD51 recombinase
16719515	7.97	6.54	2.7	0.0179	0.9999	MKI67	marker of proliferation Ki-67
16852312	6.47	5.05	2.68	0.0007	0.9999	SKA1	spindle and kinetochore associated complex subunit 1
16707468	7.79	6.39	2.65	0.0101	0.9999	KIF11	kinesin family member 11
16877019	6.32	4.92	2.65	0.0011	0.9999	RRM2	ribonucleotide reductase M2
16939960	6.32	4.91	2.64	0.0015	0.9999	KIF15	kinesin family member 15
16711501	5.1	3.7	2.63	0.0025	0.9999	IL2RA	interleukin 2 receptor, alpha
16705159	5.75	4.36	2.62	0.0031	0.9999	CDK1	cyclin-dependent kinase 1
17016366	5.01	3.64	2.58	0.0101	0.9999	HIST1H2AB	histone cluster 1, H2ab
16899982	7.06	5.69	2.58	0.0333	0.9999	LOC101928173	uncharacterized LOC101928173
16904780	5.43	4.07	2.57	0.0096	0.9999	SPC25	SPC25, NDC80 kinetochore complex component
17088390	5.7	4.34	2.56	0.0180	0.9999	ORM2; ORM1	orosomucoid 2; orosomucoid 1
16901755	5.81	4.47	2.54	0.0034	0.9999	BUB1	BUB1 mitotic checkpoint serine/threonine kinase
16725041	5.45	4.12	2.51	0.0264	0.9999	FAM111B	family with sequence similarity 111, member B
16820820	5.06	3.73	2.51	0.0242	0.9999	SNORD111	small nucleolar RNA, C/D box 111
16793225	6.51	5.19	2.5	0.0096	0.9999	DLGAP5	discs, large (Drosophila) homolog-associated protein 5
17010552	5.47	4.16	2.48	0.0064	0.9999	TTK	TTK protein kinase
16850477	8.33	7.02	2.47	0.0240	0.9999	TYMS	thymidylate synthetase
16834056	5.12	3.82	2.47	0.0249	0.9999	CDC6	cell division cycle 6
16867344	7.75	6.45	2.46	0.0026	0.9999	LRG1	leucine-rich alpha-2-glycoprotein 1
17016509	6.56	5.26	2.46	0.0281	0.9999	HIST1H3J	histone cluster 1, H3j
16850663	4.14	2.85	2.43	0.0049	0.9999	DLGAP1-AS2	DLGAP1 antisense RNA 2
16698684	5.66	4.38	2.43	0.0373	0.9999	IL10	interleukin 10
16773840	6.45	5.17	2.43	0.0096	0.9999	BRCA2	breast cancer 2, early onset
17111711	10.13	8.86	2.42	0.0177	0.9999	VSIG4	V-set and immunoglobulin domain containing 4
17024980	6.45	5.18	2.42	0.0049	0.9999	FBXO5	F-box protein 5
16707551	6.88	5.61	2.41	0.0188	0.9999	CEP55	centrosomal protein 55kDa
16799793	8.19	6.92	2.4	0.0138	0.9999	NUSAP1	nucleolar and spindle associated protein 1
16809748	4.7	3.45	2.39	0.0074	0.9999	MNS1	meiosis-specific nuclear structural 1
16922007	4.27	3.02	2.39	0.0416	0.9999	BACH1	BTB and CNC homology 1, basic leucine zipper transcription factor 1
16938296	7.06	5.81	2.38	0.0055	0.9999	SGOL1-AS1	SGOL1 antisense RNA 1
16991859	6.19	4.95	2.37	0.0200	0.9999	HMMR	hyaluronan-mediated motility receptor (RHAMM)
17016506	5.44	4.2	2.36	0.0048	0.9999	HIST1H4L	histone cluster 1, H4l
16800355	6.68	5.45	2.36	0.0208	0.9999	WDR76	WD repeat domain 76
17012582	5.6	4.36	2.36	0.0295	0.9999	ARG1	arginase 1
17072225	5.68	4.45	2.35	0.0072	0.9999	MTBP	MDM2 binding protein
16779546	4.21	2.98	2.34	0.0023	0.9999	DIAPH3	diaphanous-related formin 3
16882975	5.73	4.51	2.33	0.0009	0.9999	NCAPH	non-SMC condensin I complex subunit H
17105401	4.66	3.45	2.32	0.0028	0.9999	CENPI	centromere protein I
16979515	7.48	6.28	2.31	0.0165	0.9999	CCNA2	cyclin A2
16843567	7.48	6.28	2.31	0.0056	0.9999	CCL23	chemokine (C-C motif) ligand 23
16662648	5.6	4.4	2.3	0.0062	0.9999	CDCA8	cell division cycle associated 8
16844312	8.18	6.97	2.3	0.0227	0.9999	TOP2A	topoisomerase (DNA) II alpha
16760621	3.79	2.59	2.3	5.53E-06	0.2669	CDCA3	cell division cycle associated 3
16908307	4.62	3.41	2.3	0.0269	0.9999	CXCR1	chemokine (C-X-C motif) receptor 1
16677201	7.37	6.18	2.29	0.0294	0.9999	DTL	denticleless E3 ubiquitin protein ligase homolog (Drosophila)
16937741	6.78	5.59	2.29	0.0098	0.9999	PPARG	peroxisome proliferator-activated receptor gamma
17005865	8.27	7.09	2.27	0.0342	0.9999	HIST1H2BM	histone cluster 1, H2bm
16670387	6.84	5.68	2.24	0.0135	0.9999	HIST2H3A; HIST2H3C	histone cluster 2, H3a; histone cluster 2, H3c
16692616	6.84	5.68	2.24	0.0135	0.9999	HIST2H3A; HIST2H3C	histone cluster 2, H3a; histone cluster 2, H3c
16679411	5.55	4.39	2.23	0.0060	0.9999	EXO1	exonuclease 1
16708668	5.83	4.67	2.23	0.0185	0.9999	RPARP-AS1	RPARP antisense RNA 1
17118048	3.64	2.49	2.22	0.0486	0.9999	LOC100133299	GALI1870
16912362	5.12	3.98	2.21	0.0009	0.9999	ID1	inhibitor of DNA binding 1, dominant negative helix-loop-helix protein
17008092	6.85	5.71	2.21	0.0047	0.9999	PIM1	Pim-1 proto-oncogene, serine/threonine kinase
16912238	4.71	3.57	2.21	0.0036	0.9999	LOC100134868; LOC105372584	uncharacterized LOC100134868; uncharacterized LOC105372584
16826160	6.67	5.53	2.2	0.0098	0.9999	SHCBP1	SHC SH2-domain binding protein 1
16957951	5.18	4.06	2.18	0.0067	0.9999	POLQ	polymerase (DNA directed), theta
16849379	6.15	5.03	2.18	0.0108	0.9999	TK1	thymidine kinase 1, soluble
16911212	6.66	5.55	2.15	0.0369	0.9999	MCM8	minichromosome maintenance 8 homologous recombination repair factor
16697544	6.1	5	2.15	0.0185	0.9999	ASPM	abnormal spindle microtubule assembly
17002820	5.95	4.84	2.15	0.0027	0.9999	SH3PXD2B	SH3 and PX domains 2B
16962523	6.23	5.14	2.14	0.0435	0.9999	RPL39L	ribosomal protein L39-like
17042681	6.32	5.22	2.13	0.0199	0.9999	FAM20C	family with sequence similarity 20, member C
16951485	6.11	5.02	2.13	0.0117	0.9999	SGOL1	shugoshin-like 1 (S. pombe)
16702571	4.65	3.58	2.1	0.0139	0.9999	MCM10	minichromosome maintenance 10 replication initiation factor
17088377	4.4	3.33	2.1	0.0132	0.9999	ORM1	orosomucoid 1
16813342	6.67	5.61	2.09	0.0078	0.9999	PRC1	protein regulator of cytokinesis 1
16996545	5.9	4.83	2.09	0.0262	0.9999	DEPDC1B	DEP domain containing 1B
16695422	4.7	3.63	2.09	0.0207	0.9999	SLAMF1	signaling lymphocytic activation molecule family member 1
16755928	4.85	3.78	2.09	0.0010	0.9999	PARPBP	PARP1 binding protein
16716659	5.55	4.49	2.09	0.0052	0.9999	RBP4	retinol binding protein 4, plasma

16767851	4.34	3.29	2.08	0.0035	0.9999	E2F7	E2F transcription factor 7
17016496	8.12	7.07	2.08	0.0179	0.9999	HIST1H2AK	histone cluster 1, H2ak
16840902	5.85	4.79	2.08	0.0032	0.9999	AURKB	aurora kinase B
16809872	3.98	2.93	2.07	0.0411	0.9999	LOC101928635	uncharacterized LOC101928635
16912192	4.88	3.83	2.07	0.0171	0.9999	GINS1	GINS complex subunit 1 (Psf1 homolog)
16698023	5.65	4.6	2.07	0.0189	0.9999	UBE2T	ubiquitin conjugating enzyme E2T
17078558	7.42	6.37	2.07	0.0215	0.9999	PAG1	phosphoprotein membrane anchor with glycosphingolipid microdomains 1
16663958	4.81	3.77	2.07	0.0131	0.9999	KIF2C	kinesin family member 2C
17065652	3.82	2.77	2.06	0.0126	0.9999	MSRA	methionine sulfoxide reductase A
16871343	4.25	3.2	2.06	0.0027	0.9999	LOC101927522	uncharacterized LOC101927522
16957170	6.09	5.05	2.06	0.0045	0.9999	KIAA1524	KIAA1524
16872803	4.51	3.47	2.05	0.0106	0.9999	CEACAM1	carcinoembryonic antigen-related cell adhesion molecule 1 (biliary glycoprotein)
16913957	5.45	4.42	2.05	0.0165	0.9999	MYBL2	v-myb avian myeloblastosis viral oncogene homolog-like 2
16806757	5.84	4.81	2.05	0.0008	0.9999	FMN1	formin 1
16777278	6.47	5.44	2.04	0.0108	0.9999	SKA3	spindle and kinetochore associated complex subunit 3
16967794	5.79	4.76	2.03	0.0118	0.9999	CXCL1	chemokine (C-X-C motif) ligand 1 (melanoma growth stimulating activity, alpha)
16707695	5.46	4.44	2.03	0.0161	0.9999	HELLS	helicase, lymphoid-specific
16916958	8.09	7.07	2.02	0.0274	0.9999	PCNA	proliferating cell nuclear antigen
16697695	6.25	5.24	2.02	0.0177	0.9999	KIF14	kinesin family member 14
16666977	3.66	2.65	2.01	0.0131	0.9999	LRRC8C	leucine rich repeat containing 8 family, member C
17099397	4.21	3.21	2	0.0011	0.9999	LOC105376306	uncharacterized LOC105376306
16818451	2.51	3.54	-2.04	0.0195	0.9999	TP53TG3D	TP53 target 3D
16924101	2.91	3.95	-2.05	0.0378	0.9999	TEKT4P2	tektin 4 pseudogene 2
16658752	8.01	9.12	-2.15	0.0499	0.9999	RBP7	retinol binding protein 7, cellular
16757616	3.77	4.88	-2.17	0.0007	0.9999	MAP1LC3B2	microtubule-associated protein 1 light chain 3 beta 2
16875034	3.04	4.16	-2.17	0.0175	0.9999	ZNF836	zinc finger protein 836
16975707	6.12	7.29	-2.25	0.0091	0.9999	NFXL1	nuclear transcription factor, X-box binding-like 1
16875599	5.05	6.24	-2.28	0.0160	0.9999	TNNT1	troponin T type 1 (skeletal, slow)
16932526	2.71	3.91	-2.3	0.0015	0.9999	LOC729461; LOC105372944	uncharacterized LOC729461; uncharacterized LOC105372944
16675301	4.27	5.47	-2.3	0.0142	0.9999	RGS1	regulator of G-protein signaling 1
16900116	2.47	3.68	-2.31	0.0017	0.9999	IGKV1-9	immunoglobulin kappa variable 1-9
16820702	2.63	3.85	-2.32	0.0071	0.9999	CLEC18C; CLEC18A	C-type lectin domain family 18, member C; C-type lectin domain family 18, member A
16924878	5.3	6.7	-2.62	0.0018	0.9999	TIAM1	T-cell lymphoma invasion and metastasis 1
17005211	1.54	3	-2.74	0.0073	0.9999	MIR548A1	microRNA 548a-1
16934836	5.83	7.61	-3.44	0.0113	0.9999	LGALS2	lectin, galactoside-binding, soluble, 2
